# Supplementary material for: Arc-like magmas generated by mélange-peridotite interaction in the mantle wedge
Source: Nat Commun. 2018 Jul 20;9:2864. doi: 10.1038/s41467-018-05313-2 (PMC6054672; doi:10.1038/s41467-018-05313-2)
Supplement: Supplementary file 1 — Supplementary Information [file 41467_2018_5313_MOESM1_ESM.pdf]

Arc-like magmas generated by mélange-peridotite interaction in the mantle wedge

E. A. Codillo[1], V. Le Roux[*,2], H. R. Marschall[3]

[1]Massachusetts Institute of Technology/Woods Hole Oceanographic Institution Joint Program in Oceanography/Applied Ocean Science and Engineering, Woods Hole, Massachusetts 02543, USA
[2] Department of Geology and Geophysics, Woods Hole Oceanographic Institution, 266 Woods Hole Road, Woods Hole Massachusetts 02543 USA
[2] Institut für Geowissenschaften, Goethe Universität Frankfurt, Altenhöferalle 1, 60438 Frankfurt am Main, Germany

*corresponding author: vleroux@whoi.edu

Supplementary Figures

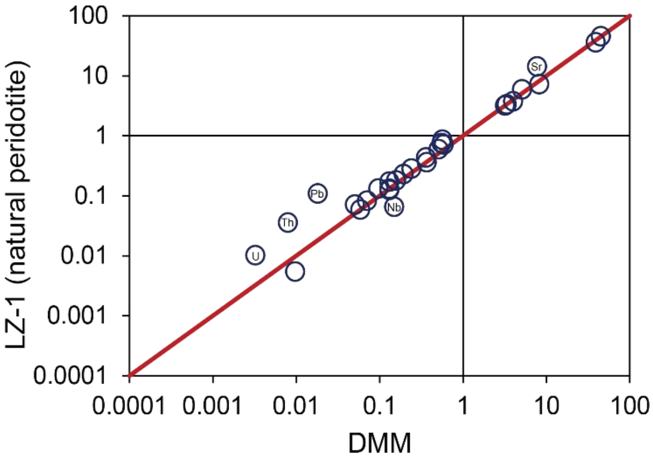

Supplementary Figure 1 | Composition of natural peridotite LZ-1 used in this study, compared with DMM composition. The plot demonstrates a close compositional similarity for major and trace elements between LZ-1 (Supplementary Dataset 1) and DMM[1] compositions. The red line is the 1:1 ratio line.

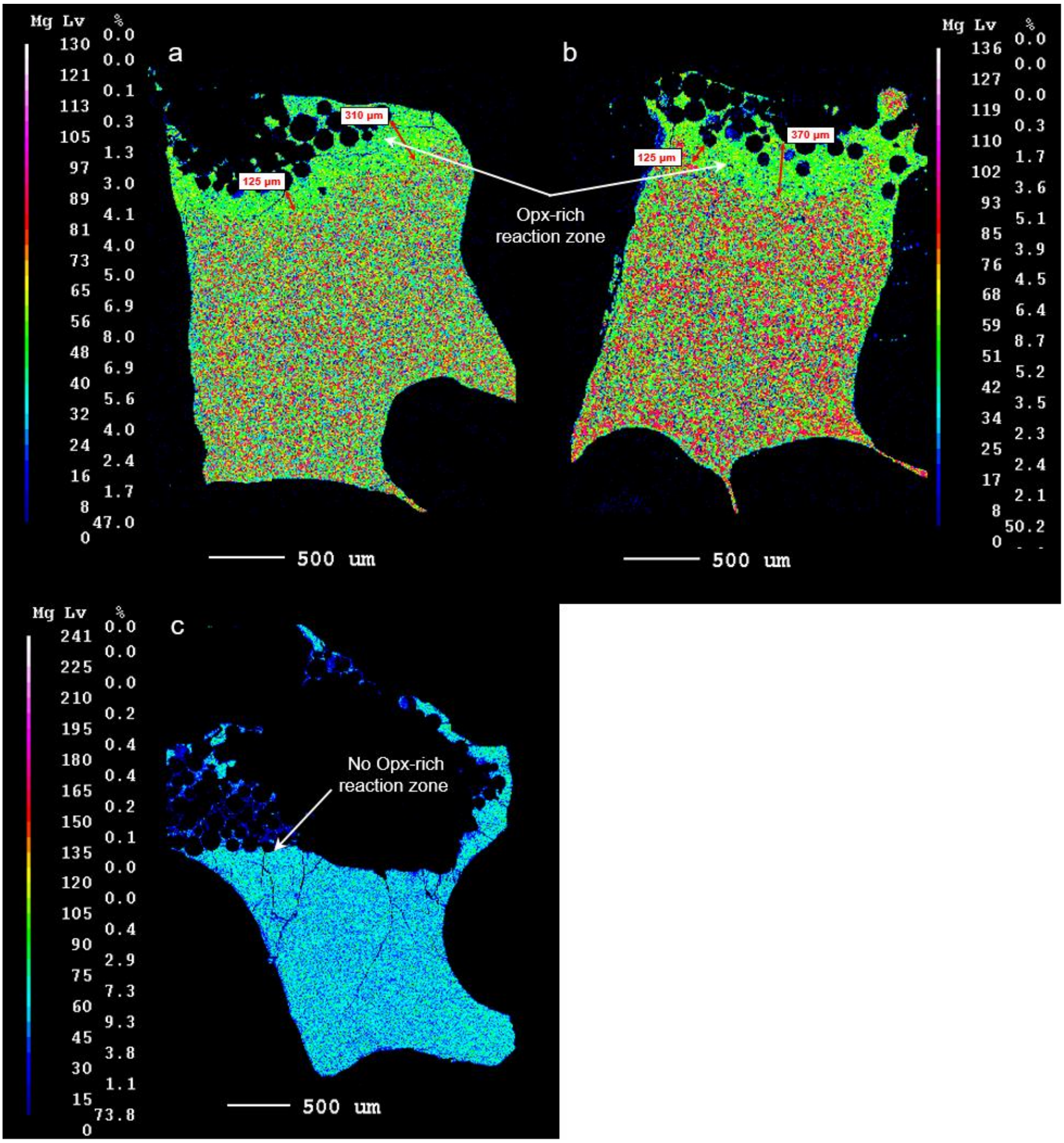

Supplementary Figure 2 | Mg compositional maps of representative experiments. Mg compositional maps of 72-h experiments performed at 1.5 GPa. (a) PER-SED (85-15) at 1280 ºC and (b) PER-SED (85-15) at 1315 ºC, showing a 125–370 µm-thick Opx-rich reaction zone (green areas). (c) No opx-rich reaction zone was observed in the 3-h experiment PER-SED (95-5) at 1.5 GPa and 1280 ºC.

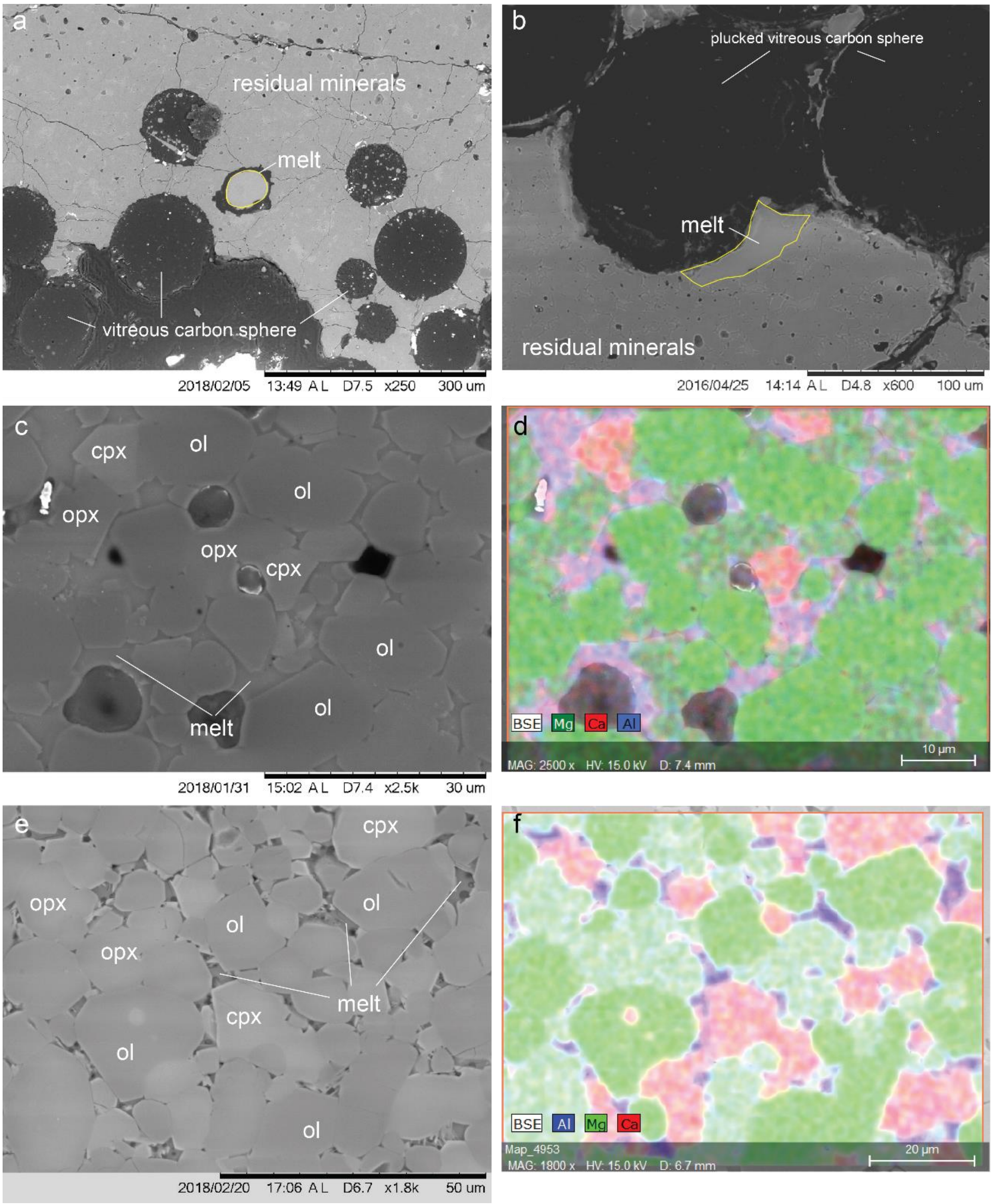

Supplementary Figure 3 | Representative BSE and EDS images of experimental run products at 1.5 GPa. Olivine (ol), Orthopyroxene (opx), Clinopyroxene (cpx) and melt are identified. Dark zones are holes/voids left by plucked out minerals during polishing. Dark round circles are polished (and sometimes plucked) vitreous carbon spheres. (a) A well-exposed circular melt pool occupying the outline of a carbon sphere in PER-SED 85-15 at 1315 ^{o}C, and (b) melt pool around a plucked carbon sphere in PER-SED 85-15 at 1280 ^{o}C. (c) BSE image and (d) Mg-Ca-Al chemical map of PER-SED 85-15 at 1280 ^{o}C highlighting the assemblage of ol + opx + cpx + melt. (e) BSE image and (f) Mg-Ca-Al chemical map of the near-solidus experiment PER-SERP 85-15 at 1230 ^{o}C highlighting the assemblage of ol + opx + cpx + minor amount of melt. Melt compositions from near-solidus experiments were not used in this study as abundant dendrites were noticed.

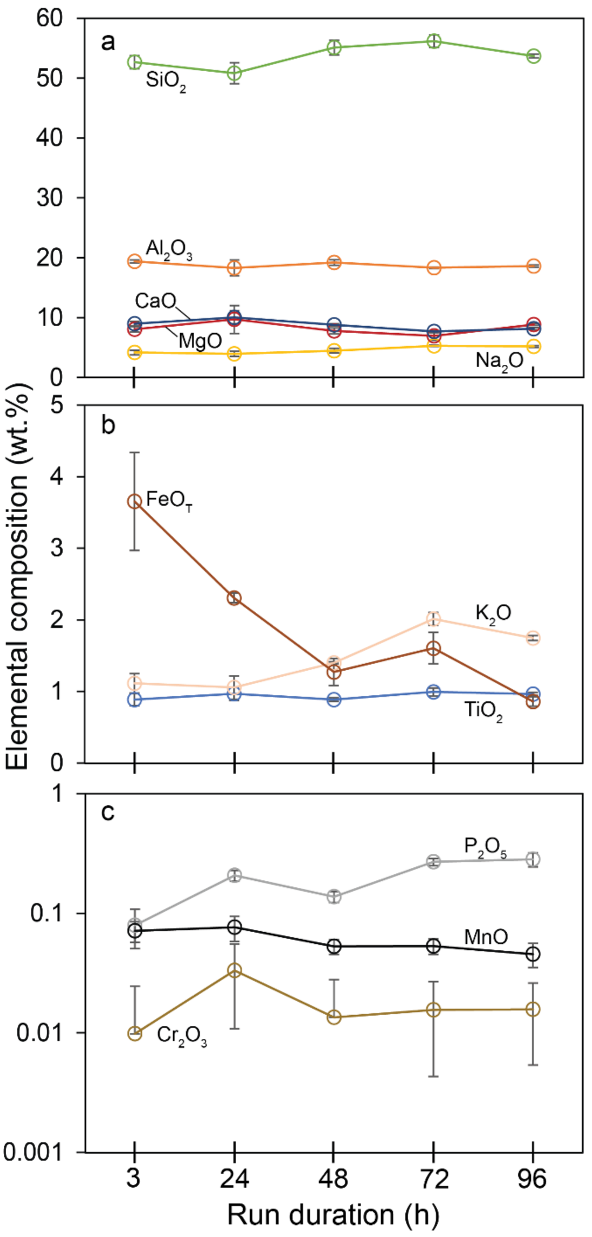

Supplementary Figure 4 | Determination of minimum run duration through a time-series experiments. Element compositional variations, (a) SiO_2, Al_2O_3, CaO, MgO, Na_2O, (b) FeO_T, K_2O, TiO_2, (c) P_2O_5, MnO, Cr_2O_3, in a time series experiments at 1.5 GPa and 1280 ºC (PER-SED 95-5; Supplementary Dataset 2), with run duration ranging from 3-h to 96-h. We chose a run duration of 72-h to ensure close approach to equilibrium. The data are plotted as averages with error bars representing 1 s.d.

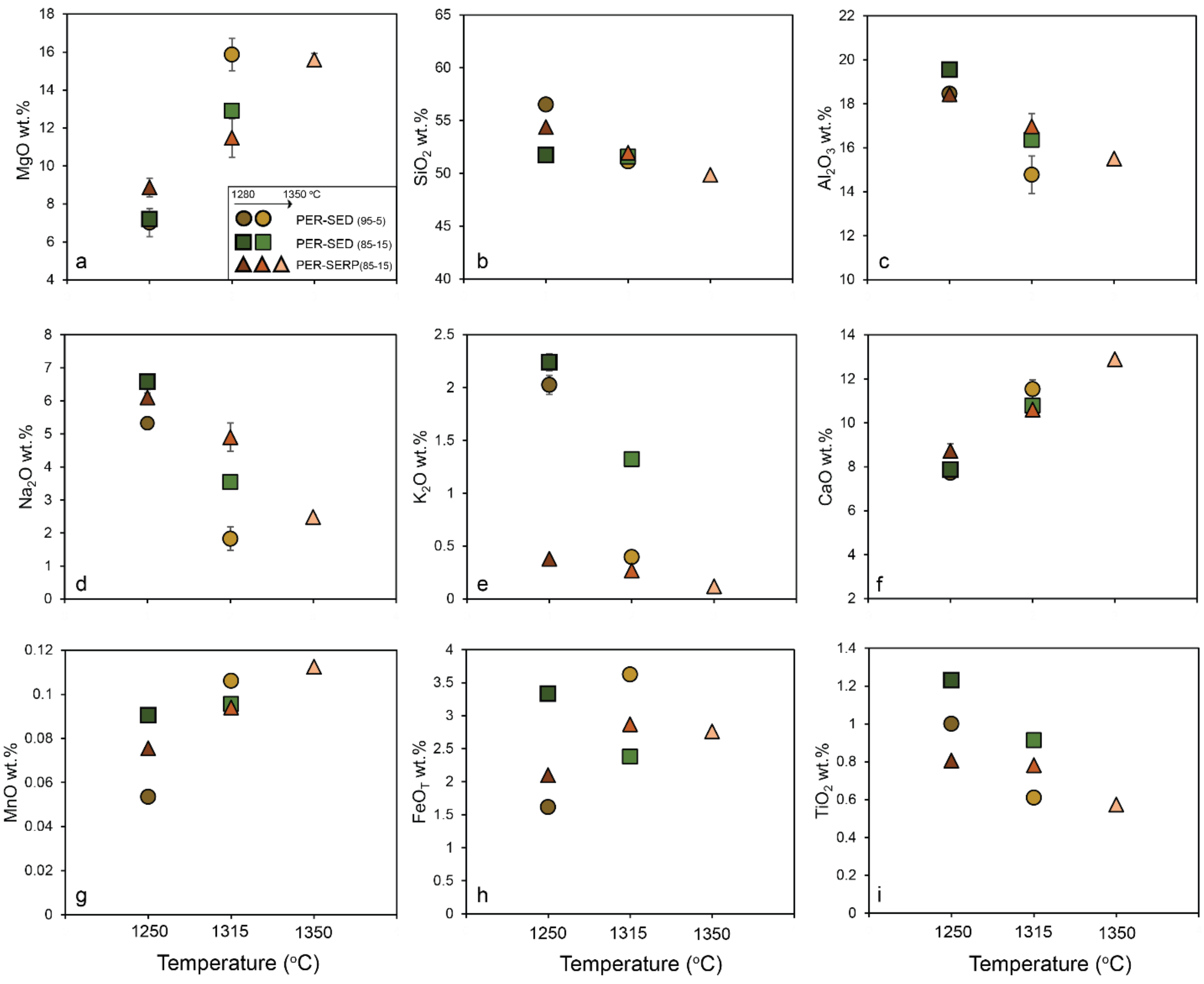

Supplementary Figure 5 | Major element variations of experimental peridotite-mélange melts with temperature. (a) MgO, (b) SiO_2, (c) Al_2O_3, (d) Na_2O, (e) K_2O, (f) CaO, (g) MnO, (h) FeO_T, (i) TiO_2 variations vs temperature (^oC). The data are plotted as averages with error bars representing 1 s.d. (Supplementary Dataset 2).

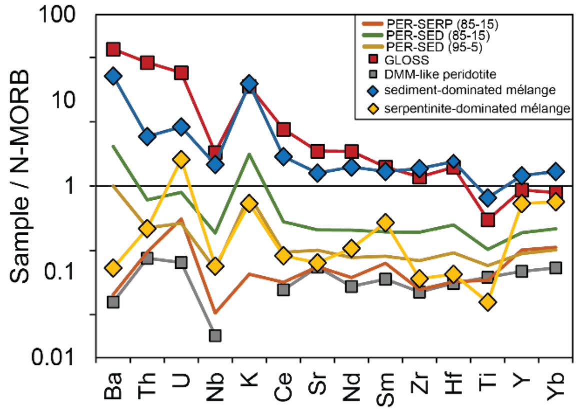

Supplementary Figure 6 | Trace element compositions of starting materials PER-SED and PER-SERP and their components. DMM-like peridotite is LZ-1, sediment-dominated mélange is SY400B and serpentine-dominated mélange is SY325 (Supplementary Dataset 1). GLOSS composition is from Plank and Langmuir[2]. The average N-MORB value used in the normalization is from Gale *et al*.[3]

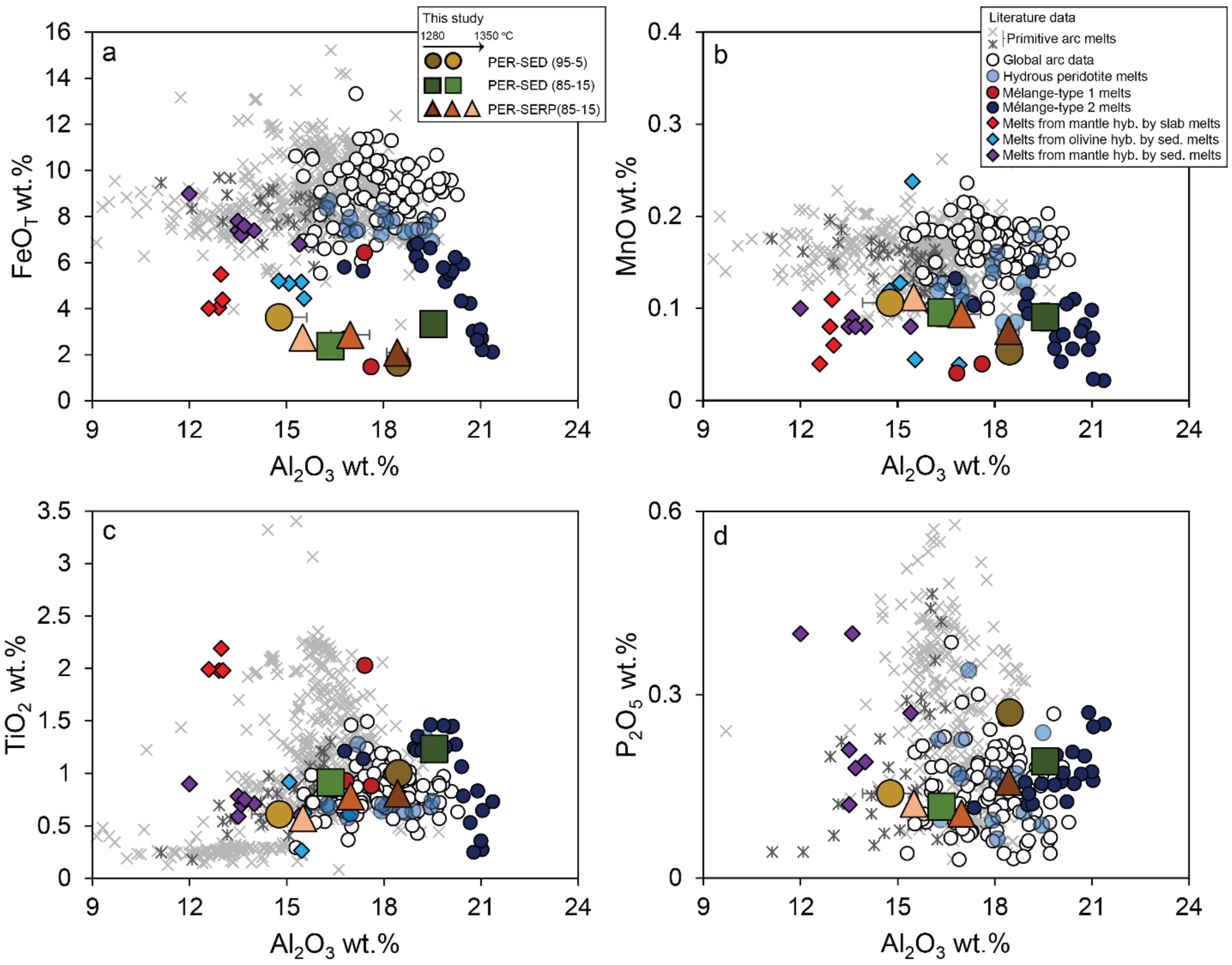

Supplementary Figure 7 | Major element composition of experimental melts. Major element variations (a) FeO_T, (b) MnO, (c) TiO_2, (d) P_2O_5 vs Al_2O_3 of experimental peridotite-mélange melts from this study compared to global arcs[4] (normalized to MgO = 6 wt. %), two primitive arc melts compilations, and previous experimental studies[5–7]. The two primitive arc melts compilations are from Schmidt and Jagoutz[8] (gray asterisk) and Till *et al.*[9] (light gray cross). Hydrous peridotite melts are from Gaetani and Grove[10]. Experimental melts from mantle hybridized by slab melts and sediment melts are from Rapp *et al.*[5] and Mallik *et al.*[7], respectively. Experimental melts of olivine hybridized by sediment melts are from Pirard and Hermann[6]. Experimental mélange-type 1 melts are from Castro and Gerya[11] and Castro *et al.*[12], while the experimental mélange-type 2 melts are from Cruz-Uribe *et al.*[13] Our experiments are plotted as averages with error bars representing 1 s.d. All the data, including the literature, are plotted on volatile-free basis.

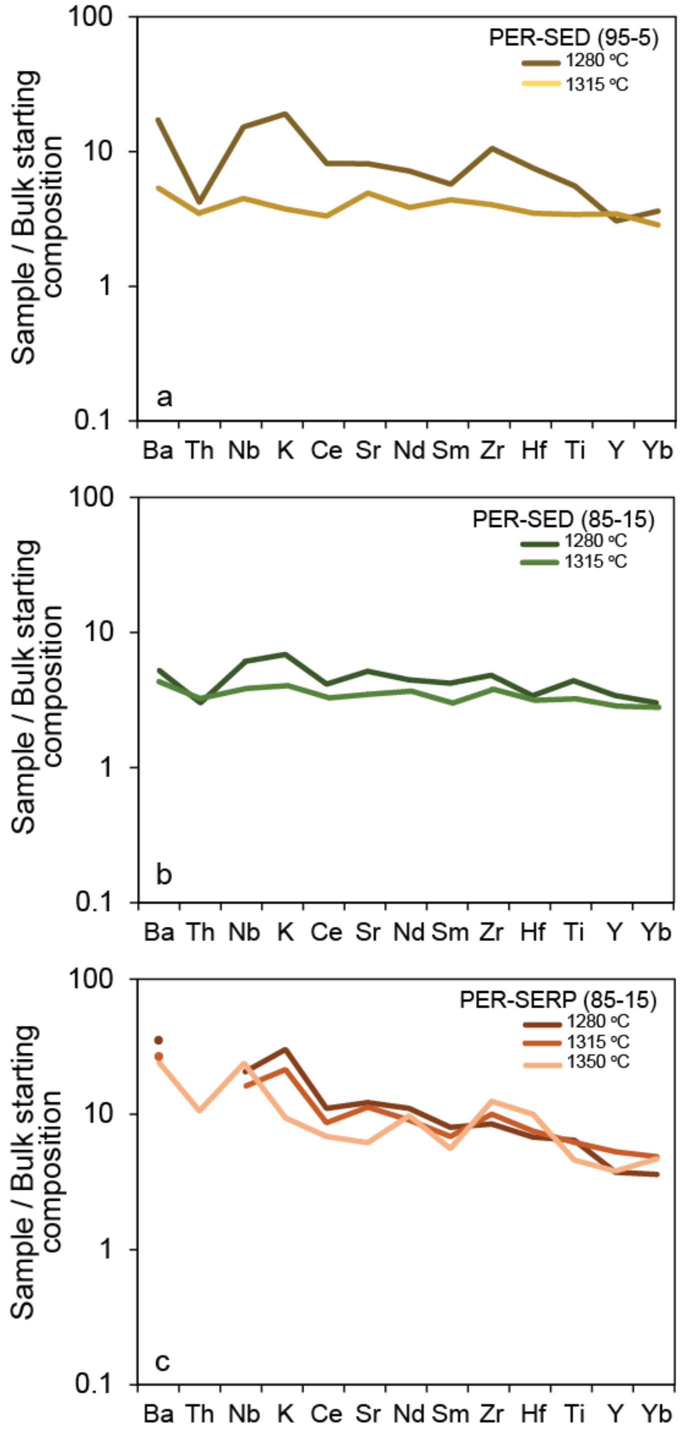

Supplementary Figure 8 | Trace element compositions of experimental peridotite-mélange melts normalized to bulk starting compositions. (a) PER-SED (95-5), (b) PER-SED (85-15) and (c) PER-SERP (85-15). The bulk starting compositions are summarized in Supplementary Dataset 1 and experimental melts compositions are reported in Supplementary Dataset 2.

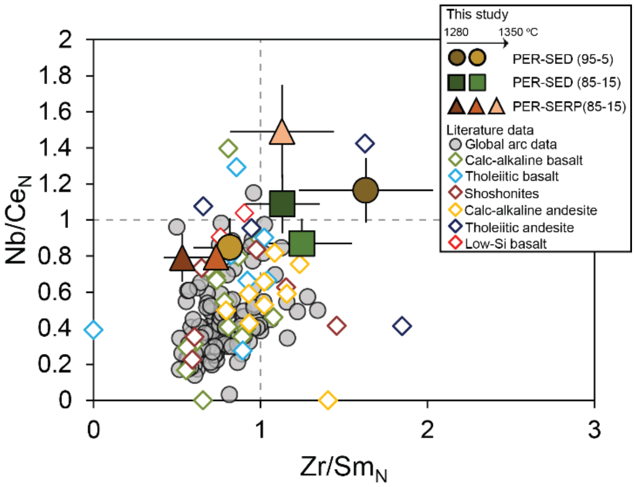

Supplementary Figure 9 | N-MORB normalized Nb/Ce versus Zr/Sm plot of experimental peridotite-mélange melts compared to arc magma compositions. Arc magma literature databases include the global arc data[4] (circle symbol; normalized to MgO = 6 wt. %) and compiled primitive arc magmas[8] (diamond symbols). N-MORB value used in normalization is from Gale *et al*.[3]

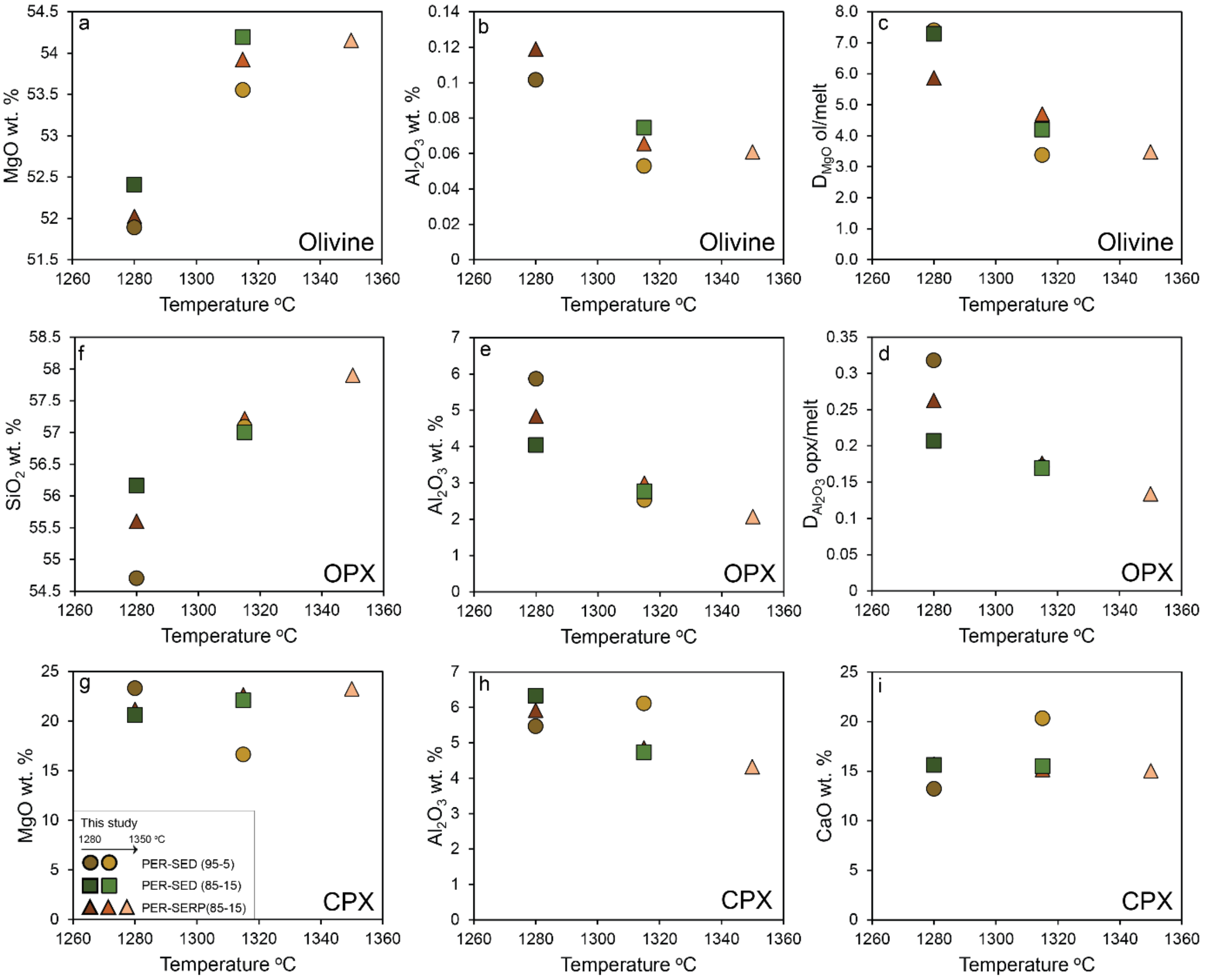

Supplementary Figure 10 | Major element and partition coefficient variations between minerals and melt with temperature. Mineral chemistry data for olivine (ol), orthopyroxene (opx) and clinopyroxene (cpx) are summarized in Supplementary Dataset 5. The data are plotted as averages.

Supplementary References

1. Workman, R. K. & Hart, S. R. Major and trace element composition of the depleted MORB mantle (DMM). *Earth Planet. Sci. Lett.* **231,** 53–72 (2005).
2. Plank, T. & Langmuir, C. H. The chemical composition of subducting sediment and its consequences for the crust and mantle. *Chem. Geol.* **145,** 325–394 (1998).
3. Gale, A., Dalton, C. A., Langmuir, C. H., Su, Y. & Schilling, J.-G. The mean composition of ocean ridge basalts. *Geochem. Geophys. Geosystems* **14,** 489–518 (2013).
4. Turner, S. J. & Langmuir, C. H. The global chemical systematics of arc front stratovolcanoes: Evaluating the role of crustal processes. *Earth Planet. Sci. Lett.* **422,** 182–193 (2015).
5. Rapp, R.P, Shimizu, N, Norman, M.D & Applegate, G.S. Reaction between slab-derived melts and peridotite in the mantle wedge: experimental constraints at 3.8 GPa. *Chem. Geol.* **160,** 335–356 (1999).
6. Pirard, C. & Hermann, J. Focused fluid transfer through the mantle above subduction zones. *Geology* **43,** 915–918 (2015).
7. Mallik, A., Nelson, J. & Dasgupta, R. Partial melting of fertile peridotite fluxed by hydrous rhyolitic melt at 2–3 GPa: implications for mantle wedge hybridization by sediment melt and generation of ultrapotassic magmas in convergent margins. *Contrib. Mineral. Petrol.* **169,** 48 (2015).
8. Schmidt, M. W. & Jagoutz, O. The global systematics of primitive arc melts. *Geochem. Geophys. Geosystems* **18,** 2817–2854 (2017).
9. Till, C. B. A review and update of mantle thermobarometry for primitive arc magmas. *Am. Mineral.* **102,** 931 (2017).
10. Gaetani, G. A. & Grove, T. L. The influence of water on melting of mantle peridotite. *Contrib. Mineral. Petrol.* **131,** 323–346 (1998).
11. Castro, A. & Gerya, T. V. Magmatic implications of mantle wedge plumes: Experimental study. *Lithos* **103,** 138–148 (2008).

12. Castro, A. *et al.* Melting Relations of MORB–Sediment Mélanges in Underplated Mantle Wedge Plumes; Implications for the Origin of Cordilleran-type Batholiths. *J. Petrol.* **51,** 1267–1295 (2010).

13. Cruz-Uribe, A. M., Marschall, H. R., Gaetani, G. A. & Le Roux, V. Generation of alkaline magmas in subduction zones by partial melting of mélange diapirs—An experimental study. *Geology* **46,** 343–346 (2018).
