# Supplementary material for: Arc-like magmas generated by mélange-peridotite interaction in the mantle wedge
Source: Nat Commun. 2018 Jul 20;9:2864. doi: 10.1038/s41467-018-05313-2 (PMC6054672; doi:10.1038/s41467-018-05313-2)
Supplement: Supplementary file 3 — Description of Additional Supplementary Files [file 41467_2018_5313_MOESM3_ESM.pdf]

Description of Additional Supplementary Files

File Name: Supplementary Data 1
Description: Major and trace element compositions of starting materials.

File Name: Supplementary Data 2
Description: Major and trace element compositions of experimental peridotite-mélange melts.

File Name: Supplementary Data 3
Description: Summary of experimental set-up.

File Name: Supplementary Data 4
Description: Compiled major and trace element compositions of experimental melts from other studies on mantle wedge hybridization.

File Name: Supplementary Data 5
Description: Major element composition of mineral phases analyzed by EPMA.
